# *CLEC16A* variants conferred a decreased risk to allergic rhinitis in the Chinese population

**DOI:** 10.3389/fgene.2022.1053761

**Published:** 2022-12-12

**Authors:** Yongliang Niu, Haiying Wang, Zhengqing Li, Bilal Haider Shamsi, Mingxia Liu, Juan Liu, Qiang Wang, Yonglin Liu

**Affiliations:** ^1^ Department of Respiratory and Critical Care Medicine, Shenmu Hospital, The Affiliated Shenmu Hospital of Northwest University, Shenmu, China; ^2^ Department of Science and Education, Shenmu Hospital, The Affiliated Shenmu Hospital of Northwest University, Shenmu, China; ^3^ Department of Cardiovascular Medicine, Shenmu Hospital, The Affiliated Shenmu Hospital of Northwest University, Shenmu, China; ^4^ Department of Prevention and Protection, Shenmu Hospital, The Affiliated Shenmu Hospital of Northwest University, Shenmu, China; ^5^ President Office, Shenmu Hospital, The Affiliated Shenmu Hospital of Northwest University, Shenmu, China

**Keywords:** allergic rhinitis, CLEC16A, variants, association analysis, Chinese population

## Abstract

**Background:** Allergic rhinitis (AR) is a chronic respiratory disease. Hereditary factors played a key role in the pathogenesis of the AR. This study investigated the association between *CLEC16A* variants and AR risk in the Chinese population.

**Methods:** We applied Agena MassARRAY technology platform to genotype five single nucleotide polymorphisms (SNPs) located in *CLEC16A* in 1004 controls and 995 cases. The association between *CLEC16A* SNPs (rs2286973, rs887864, rs12935657, rs11645657 and rs36045143) and AR risk were calculated by logistic regression analysis, with odds ratio (OR) and 95% confidence interval (CI). False-positive report probability (FPRP) was also used to assess the significant results to reduce false positives. Multifactor dimensionality reduction (MDR) was completed to assess the interaction between *CLEC16A* variants to predict AR risk.

**Results:** Totally, *CLEC16A* (rs887864, rs12935657, rs2286973, rs11645657 and rs36045143) were significantly associated with AR risk. Therein, rs2286973, rs11645657 and rs36045143 were related to a decreased risk of AR in the people ≤ 43 years old, females and the people with BMI≤24, respectively. And rs887864 and rs12935657 were also associated with a decreased susceptibility of AR in the people >43 years old. Meanwhile, in the results of region stratification, rs887864 conferred a reduced risk to AR in the people from loess hilly area.

**Conclusion:** *CLEC16A* variants conferred a decreased risk to AR in the Chinese population.

## Introduction

Allergic rhinitis (AR) is a chronic respiratory disease characterized by allergen sensitization mainly mediated by immunoglobulin E (IgE). It is characterized by runny nose, sneezing, stuffy nose, pruritus and other triggers. Several factors have been implicated in the etiology of AR, including pollen, dust mites, mold, animal dander and other factors (Skoner, 2001). The disease has a major impact on patients’ quality of life and healthcare spending (Canonica et al., 2008). Globally, AR affects approximately 10%–30% of the population. And due to genetic susceptibility, and environmental exposure, it was steadily increasing in the world (Passali et al., 2018). Although the etiology of AR has not been elucidated, there was sufficient evidence that cytokines played an important role in the development and process of AR (Sio et al., 2022).

*CLEC16A* (*C-Type Lectin Domain Family 16 Member A*) was formerly known as *KIAA0350.* It is located in the chromosome 16p13.13. This gene encodes a family member containing a C-type lectin domain. The protein CLEC16A was shown to be a cytosolic protein that binds to Vps16A and regulates the receptor expression through autophagy. In a CLEC16A knockout mice through Cre/loxP system, they found up-regulation of cytokine and chemokine secretion, imbalance of dendritic cell subsets and change of receptor expression in mice (Pandey et al., 2019). Some researchers also found that the deletion of CLEC16A leaded to the increase of Nrdp1 targeting parkin (Soleimanpour et al., 2014), and Golgi-related CLEC16A negatively regulated autophagy by regulating mTOR pathway (Tam et al., 2017). At present, CLEC16A was recognized as a susceptibility gene for autoimmune diseases, such as multiple sclerosis (Mero et al., 2011), juvenile idiopathic arthritis and rheumatoid arthritis (Skinningsrud et al., 2010). This suggested that CLEC16A may be the main regulator of abnormal autoimmune response.

Single nucleotide polymorphisms (SNPs) were reported to be an important genetic factor in abnormal autoimmune response. In one previous study, Hakonarson et al. identified CLEC16A as a protective gene for type 1 diabetes gene (Hakonarson et al., 2007). Also, Hirschfield et al. observed the association between primary biliary cirrhosis and variants of immune regulatory gene *CLEC16A* (Hirschfield et al., 2012). Besides, *CLEC16A* polymorphisms may involve in pathogenesis of allergic diseases (Revez et al., 2016; Ashley et al., 2017), which can increase or decrease susceptibility to AR through interleukin, and other genes (Black et al., 2009). In an article reported by Ferreira et al., they observed *CLEC16A* rs62026376 associated with increased asthma with hay fever risk (Ferreira et al., 2014). So, it may be a risky variant. However, *CLEC16A* variants have been rarely reported in AR population from China.

In the present study, we selected five variants of *CLEC16A* (rs2286973, rs887864, rs12935657, rs11645657 and rs36045143) based on the 1000 genome project. Based on the NCBI website, we found that rs887864, rs12935657, rs11645657 and rs36045143 were located in the intron region of *CLEC16A*, while rs2286973 was located in the last exon region of *CLEC16A*. When rs2286973 G mutates into rs2286973 A, the encoded amino acid has not changed. Subsequently, we assessed the relationship between *CLEC16A* variants (rs2286973, rs887864, rs12935657, rs11645657 and rs36045143) with AR risk in the Han population from Shaanxi, China.

## Materials and methods

### Study population

The study was approved by the Ethics Committee of Shenmu Hospital and the authorization number was sm004. In this study, all the involved subjects were the Chinese Han population. Totally, 1004 controls (346 males and 658 females) and 995 patients with AR (371 males and 624 females) were consecutively recruited from 5 towns (Daliuta Town in plain lands area, Jinjie Town, Langanbao Town, Hejiachuan Town and central urban area in hilly areas) in Shenmu city. The selected criteria for patients with AR were as follows. ①Clinical symptoms: the patient has two or more symptoms such as sneezing, runny nose, itchy nose, blocked nose and so on. ②Nasal symptoms: pale and swollen bilateral nasal mucosa, inferior turbinate edema, and watery nasal discharge. ③Allergen testing: Blood was drawn to examine specific IgE, and the diagnosis of AR was confirmed if the patient had high specific IgE in serum. The exclusion criteria for AR patients were: no history of asthma, no comprehensive disease such as lung cancer, and no infectious disease such as tuberculosis. Also, based on the routine physical examination, the controls had no history of respiratory diseases, including sinusitis, rhinitis and other inflammatory disease. By reviewing the participant’s hospital records, we collected the information of participants, such as age, sex, height, weight, place of origin, residence, occupation, past medical history, family medical history, etc. In addition, we also collected the blood routine indicators obtained by the blood analyzer, such as red blood cell distribution width_SD (RDW_SD), basophil count (BASO), eosinophil count (EO), hemoglobin (HGB), mean hemoglobin concentration (MCHC), neutrophil ratio (NEUT_per), lymphocyte ratio (LYMPH_per), eosinophil ratio (EO_per), basophil ratio (BASO_per) and red blood cell distribution width_CV (RDW_CV). Biochemical indicators (fasting blood glucose, FBG) and renal function indicators (uric acid, UA) were measured by drawing venous blood. Written informed consent was collected from all subjects. All procedures comply with the regulations of the Department of Health and Human Services (DHHS) on the protection of human research objects.

### Genotyping of SNPs

According to the SNPs data downloaded from the 1000 genome project (http://www.internationalgenome.org/), *CLEC16A* variants (rs2286973, rs887864, rs12935657, rs11645657 and rs36045143) with minor allele frequencies> 5% and Hardy-Weinberg equilibrium (HWE) > 0.01 were chosen in the global population. According to the specificity principle of primers, these sites meet the requirements. The SNP-related primers (amplification and extension primers) were finished and listed in Supplementary Table S1. 3ml peripheral blood samples were collected from participants in EDTA pretreated tubes. Subsequently, genomic DNA extracted by the GoldMag Whole Blood Genomic DNA Purification Kit (GoldMag Co. Ltd., Xi’an, China), which was used as the PCR amplification template. Subsequently, DNA concentration was estimated by NanoDrop 2000 (Thermo Scientific, Waltham, Massachusetts, United States). Then, using the Agena MassARRAY platform with iPLEX gold chemistry (Agena Bioscience, San Diego, CA, United States), genotyping of *CLEC16A* variants were proceeded in a 384-well plate. And data management was performed by Agena Bioscience TYPER software, Version 4.0 (Ding et al., 2015).

### Statistical analysis

IBM SPSS statistical software version 24 and Microsoft Excel were used to analyze the socio demographic descriptive data of the case group and the control group. The continuous variables were expressed as mean ± standard error (SE) and compared by Student’s t-test. Age and sex distribution differences of AR patients and controls were assessed by *t*-test and chi-square test, respectively. Meanwhile, in the controls, we analyzed whether the selected SNP sites met the Hardy-Weinberg equilibrium by chi-square test. Chi-square test was also used to analyze the genotype and allele distribution of AR patients and controls. The relationship between *CLEC16A* variants and AR risk was assessed by logistic regression analysis calculated by the PLINK software, version 1.07 (Harvard, Boston, MA, United States). Generally, OR and 95% CI were calculated by logistic regression to analyze the association under four genetic models (codominant, dominant, recessive and log-additive models). To further eliminate false positive in the data, we used FPRP to analyze the significant results. Multifactor dimensionality reduction (MDR) was completed to assess the interaction between *CLEC16A* variants to predict AR risk. *P* less than 0.05 was considered as significative.

## Results

### The basic information of study population and the selected variants

Noteworthy, 1004 controls and 995 patients with AR were collected in this study. The mean age of the 1004 controls (346 males and 658 females) and 995 patients (371 males and 624 females) were 43.77 ± 0.26 years old and 42.81 ± 0.33 years old, respectively (Table 1). There was a difference in age between the two groups (*p* = 0.002), but there was no difference in sex and BMI between the two groups (*p* = 0.188, *p* = 0.796, respectively). In addition, there was no significant difference between the two groups in the distribution of wind beach area and loess hilly area (*p* = 0.738). Supplementary Table S2 listed the clinical parameters of cases and controls.

**TABLE 1:** Basic information of patients with AR and controls.

Variable	Mean ± SE		*p*-value
	Control (N = 1004)	Case (N = 995)	
Age (years)	43.77 ± 0.26	42.81 ± 0.33	0.002^a^
≤43	431 (42.9%)	488 (49.0%)	
>43	573 (57.1%)	507 (51.0%)	
Sex			
Male	346 (34.5%)	371 (37.3%)	0.188^b^
Female	658 (65.5%)	624 (62.7%)	
BMI (kg/m^2^)	24.87 ± 0.12	24.83 ± 0.11	0.796^a^
≤24	474 (47.2%)	490 (49.2%)	
>24	530 (52.8%)	505 (50.8%)	
Region			
Wind beach area	270 (26.9%)	261 (26.2%)	0.738^b^
Loess hilly area	734 (73.1%)	734 (73.8%)	

AR: Allergic rhinitis; SE: Standard error.

*p* ^a^-value was calculated by Student’s *t*-test.

*p* ^b^-value was calculated by Pearson's χ^2^ test.

In Table 2, the basic information of *CLEC16A* variants (rs2286973, rs887864, rs12935657, rs11645657 and rs36045143) were listed, including SNP-ID, gene, chromosome, position, localization, allele, minor allele frequency, major allele frequency and HWE *p*-value. The genotype distribution of all loci in the control group met Hardy-Weinberg equilibrium. We listed the function of the selected SNPs predicated by Regulome DB scores and HaploReg. And the primers of *CLEC16A* variants were shown in Supplementary Table S1.

**TABLE 2:** Basic information of *CLEC16A* variants.

SNP-ID	Gene	Chr: Position	Localization	Allele	Minor allele frequency		Major allele frequency		HWE	RegulomeDB	HaploReg
					Case	Control	Case	Score	*p*-value		
rs2286973	*CLEC16A*	16:11060913	Exon	A/G	0.324	0.346	0.676	0.654	0.727	5	DNAse, Motifs changed, Selected eQTL hits
rs887864	*CLEC16A*	16:11065028	Intron	G/A	0.220	0.247	0.780	0.753	0.498	5	DNAse, Motifs changed, Selected eQTL hits
rs12935657	*CLEC16A*	16:11125184	Intron	A/G	0.095	0.108	0.905	0.893	0.620	1f	DNAse, Selected eQTL hits
rs11645657	*CLEC16A*	16:11129597	Intron	G/C	0.297	0.312	0.703	0.688	0.378	2b	DNAse, Motifs changed
rs36045143	*CLEC16A*	16:11131109	Intron	G/A	0.100	0.117	0.900	0.883	0.760	4	DNAse, Motifs changed

SNP: Single nucleotide polymorphism, Chr: Chromosome, HWE: Hardy-Weinberg equilibrium.

HWE *p*-value was calculated by Pearson chi-square test.

1f indicates that the variant is likely to affect binding and linked to expression of a gene target.

2b indicates that the variant is likely to affect binding.

4 indicates that the variant has minimal binding evidence.

5 indicates that the variant has minimal binding evidence.

Also, we conducted clinical parameter analysis of different genotype carriers (Supplementary Table S3). The results showed that the EO content of the patients with the three genotypes (A/A, G/A, G/G) was significantly different (*p* = 0.023). As for rs11645657, controls with the three genotypes (A/A, G/A, G/G) had different clinical parameters-HGB and UA (*p* = 0.007, *p* = 0.028), whereas, cases with A/A, G/A, G/G genotype had significantly different LYMPH_per (*p* = 0.035).

### The correlation between *CLEC16A* variants and AR risk was analyzed in the overall population

In order to analyze the above differences, we used logistic regression to evaluate the association of *CLEC16A* variants (rs2286973, rs887864, rs12935657, rs11645657 and rs36045143) with AR risk (Table 3). The results of the allele model showed that rs887864-A conferred a remarkable decreased risk to AR (adjusted OR: 0.86, 95%CI: 0.74–0.99, *p* = 0.040), while rs12935657-G had no significant association with AR risk (adjusted OR: 0.87, 95%CI: 0.71–1.07, *p* = 0.197). In the results of genetic models (codominant, dominant, recessive and log-additive) analysis, rs887864 was significantly associated with AR risk (codominant-A/A genotype: adjusted OR: 0.60, 95%CI: 0.40–0.91, *p* = 0.016; recessive-G/G-G/A genotype: adjusted OR: 0.62, 95%CI: 0.41–0.93, *p* = 0.021; adjusted OR: 0.86, 95%CI: 0.74–0.99, log-additive: *p* = 0.043). The above results illustrated that allele-A and genotype-AA may play a protective role in the pathogenesis of AR. And, a significant correlation between rs12935657 and AR susceptivity was observed in the codominant (G/G genotype: OR: 0.24, 95%CI: 0.07–0.83, *p* = 0.025) and recessive (G/G genotype: OR: 0.24, 95%CI: 0.07–0.84, *p* = 0.025) models, suggesting that genotype-GG may play a protective role in the pathogenesis of AR. We also did the FPRP analysis for the significant associations of *CLEC16A* variants with AR risk (Supplementary Table S4). When the statistical power assumed to be 1.00, the noteworthy association between rs887864 and AR risk in the allele, codominant, recessive and log-additive models (FPRP = 0.097, 0.136 and 0.147, respectively), with noteworthiness for OR of 1.50/0.67 and the FPRP cut-off value 0.2.

**TABLE 3:** Association between *CLEC16A* variants and AR risk in the total population.

SNP-ID	Model	Genotype	Frequency		With adjustment	
			Case	Control	OR (95% CI)	*p*-value
rs2286973	Allele	A	638 (32.4%)	692 (34.6%)	1	
		G	1334 (67.6%)	1306 (65.4%)	0.90 (0.79–1.03)	0.128
	Codominant	A/A	99 (10.1%)	117 (11.7%)	1	
		G/A	440 (44.6%)	458 (45.9%)	0.91 (0.76–1.10)	0.326
		G/G	447 (45.3%)	424 (42.4%)	0.81 (0.60–1.09)	0.158
	Dominant	A/A	99 (10.1%)	117 (11.7%)	1	
		G/A-G/G	887 (89.9%)	882 (88.3%)	0.89 (0.74–1.06)	0.196
	recessive	A/A-G/A	539 (54.7%)	575 (57.6%)	1	
		G/G	447 (45.3%)	424 (42.4%)	0.85 (0.64–1.12)	0.246
	log-additive	−	−	−	0.90 (0.79–1.03)	0.130
rs887864	Allele	G	435 (22.0%)	495 (24.7%)	1	
		A	1545 (78.0%)	1507 (75.3%)	0.86 (0.74–0.99)	0.040^*^
	codominant	G/G	40 (4.0%)	65 (6.5%)	1	
		G/A	355 (35.9%)	365 (36.5%)	0.93 (0.77–1.12)	0.442
		A/A	595 (60.1%)	571 (57.0%)	0.60 (0.40–0.91)	0.016^*^
	dominant	G/G	40 (4.0%)	65 (6.5%)	1	
		G/T-A/A	950 (96.0%)	936 (93.5%)	0.88 (0.74–1.05)	0.164
	recessive	G/G-G/A	395 (39.9%)	430 (43.0%)	1	
		A/A	595 (60.1%)	571 (57.0%)	0.62 (0.41–0.93)	0.021^*^
	log-additive	−	−	−	0.86 (0.74–0.99)	0.043^*^
rs12935657	Allele	A	189 (9.5%)	215 (10.8%)	1	
		G	1797 (90.5%)	1785 (89.2%)	0.87 (0.71–1.07)	0.197
	codominant	A/A	3 (0.3%)	13 (1.3%)		
		G/A	183 (18.4%)	189 (18.9%)	0.97 (0.77–1.22)	0.786
		G/G	807 (81.3%)	798 (79.8%)	0.24 (0.07–0.83)	0.025^*^
	dominant	A/A	3 (0.3%)	13 (1.3%)	1	
		G/A-G/G	990 (99.7%)	987 (98.7%)	0.92 (0.74–1.15)	0.478
	recessive	A/A-G/A	186 (18.7%)	202 (20.2%)	1	
		G/G	807 (81.3%)	798 (79.8%)	0.24 (0.07–0.84)	0.025^*^
	log-additive	−	−	−	0.88 (0.72–1.09)	0.238
rs11645657	Allele	G	590 (29.7%)	627 (31.2%)	1	
		C	1398 (70.3%)	1381 (68.8%)	0.93 (0.81–1.06)	0.288
	codominant	G/G	90 (9.1%)	104 (10.4%)	1	
		G/C	410 (41.2%)	419 (41.7%)	0.96 (0.79–1.15)	0.636
		C/C	494 (49.7%)	481 (47.9%)	0.85 (0.62–1.16)	0.301
	dominant	G/G	90 (9.1%)	104 (10.4%)	1	
		G/C-C/C	904 (90.9%)	900 (89.6%)	0.93 (0.78–1.12)	0.453
	recessive	G/G-G/C	500 (50.3%)	523 (52.1%)	1	
		C/C	494 (49.7%)	481 (47.9%)	0.87 (0.64–1.17)	0.347
	log-additive	−	−	−	0.93 (0.82–1.07)	0.320
rs2286973	Allele	A	638 (32.4%)	692 (34.6%)	1	
		G	1334 (67.6%)	1306 (65.4%)	0.90 (0.79–1.03)	0.128
	codominant	A/A	99 (10.1%)	117 (11.7%)	1	
		G/A	440 (44.6%)	458 (45.9%)	0.91 (0.76–1.10)	0.326
		G/G	447 (45.3%)	424 (42.4%)	0.81 (0.60–1.09)	0.158
	dominant	A/A	99 (10.1%)	117 (11.7%)	1	
		G/A-G/G	887 (89.9%)	882 (88.3%)	0.89 (0.74–1.06)	0.196
	recessive	A/A-G/A	539 (54.7%)	575 (57.6%)	1	
		G/G	447 (45.3%)	424 (42.4%)	0.85 (0.64–1.12)	0.246
	log-additive	−	−	−	0.90 (0.79–1.03)	0.130

AR: Allergic rhinitis; SNP: Single nucleotide polymorphism; OR: Odds ratio; CI: Confidence interval.

^*^ Means significant difference.

*p*-value was calculated by logistic regression adjusted by age, sex and BMI.

### Effect of *CLEC16A* variants on AR risk stratified by age, sex and BMI

We further analyzed the relationship between *CLEC16A* loci and the risk of AR stratified by age, sex and BMI (Table 4 and Table 5). In Table 4, the results of age stratification showed that rs2286973, rs11645657 and rs36045143 were related to a decreased risk of AR in the people ≤43 years old (rs2286973: codominant-G/A genotype: *p* = 0.013; dominant-G/A-G/G genotype: *p* = 0.013; log-additive: *p* = 0.040; rs11645657: allele-C: *p* = 0.033; dominant-G/C-C/C genotype: *p* = 0.031; log-additive: *p* = 0.025; rs36045143: allele-A: *p* = 0.000; codominant-G/A genotype: *p* = 0.000; dominant-G/A-A/A genotype: *p* = 0.000; log-additive: *p* = 0.000). Rs887864 and rs12935657 were also associated with a decreased susceptibility of AR in the people >43 years old (rs887864: codominant-A/A genotype: *p* = 0.004; recessive-A/A genotype: *p* = 0.002; rs12935657: codominant-G/G genotype: *p* = 0.045; recessive-G/G genotype: *p* = 0.039) and the people ≤43 years old (rs887864: allele-A: *p* = 0.015; codominant-A/G genotype: *p* = 0.003; dominant-G/A-A/A genotype: *p* = 0.002; log-additive: *p* = 0.004; rs12935657: allele-G: *p* = 0.001; codominant-G/A genotype: *p* = 0.002; dominant-G/G genotype: *p* = 0.001; log-additive: *p* = 0.001) adjusted by age, sex and BMI.

**TABLE 4:** Association between *CLEC16A* variants and AR risk stratified by age.

SNP-ID	Model	Genotype	>43		≤43	
			OR (95% CI)	*p*-value	OR (95% CI)	*p*-value
rs2286973	Allele	A	1	0.794	1	
		G	0.98 (0.82–1.17)		0.83 (0.68–1.00)	0.054
	codominant	A/A	1		1	
		G/A	1.12 (0.86–1.45)	0.417	0.70 (0.52–0.93)	0.013^*^
		G/G	0.78 (0.51–1.20)	0.260	0.76 (0.48–1.20)	0.241
	dominant	A/A	1		1	
		G/A-G/G	1.04 (0.81–1.34)	0.747	0.71 (0.54–0.93)	0.013^*^
	recessive	A/A-G/A	1		1	
		G/G	0.74 (0.50–1.11)	0.143	0.91 (0.59–1.41)	0.680
	log-additive	−	0.96 (0.79–1.15)	0.651	0.81 (0.66–0.99)	0.040^*^
rs887864	Allele	G	1	0.618	1	
		A	0.95 (0.78–1.16)		0.76 (0.61–0.95)	0.015^*^
	codominant	G/G	1		1	
		G/A	1.20 (0.92–1.56)	0.171	0.64 (0.48–0.86)	0.003^*^
		A/A	0.42 (0.24–0.76)	0.004	0.67 (0.35–1.28)	0.225
	dominant	G/G	1		1	
		G/A-A/A	1.05 (0.82–1.35)	0.701	0.65 (0.49–0.85)	0.002^*^
	recessive	G/G-G/A	1		1	
		A/A	0.40 (0.22–0.70)	0.002	0.79 (0.42–1.49)	0.467
	log-additive	−	0.91 (0.74–1.12)	0.356	0.71 (0.57–0.90)	0.004^a^
rs12935657	Allele	A	1	0.226	1	
		G	1.18 (0.90–1.56)		0.60 (0.43–0.82)	0.001^*^
	codominant	A/A	1		1	
		G/A	1.37 (1.00–1.86)	0.047	0.57 (0.40–0.82)	0.002^*^
		G/G	0.11 (0.01–0.95)	0.045	0.29 (0.06–1.47)	0.135
	Dominant	A/A	1		1	
		G/A-G/G	1.27 (0.94–1.73)	0.119	0.55 (0.39–0.79)	0.001^*^
	Recessive	A/A-G/A	1		1	
		G/G	0.10 (0.01–0.89)	0.039	0.32 (0.06–1.62)	0.168
	log-additive	−	1.16 (0.87–1.55)	0.315	0.57 (0.41–0.79)	0.001^*^
rs11645657	Allele	G	1	0.552	1	
		C	1.06 (0.88–1.27)		0.80 (0.66–0.98)	0.033^*^
	codominant	G/G	1		1	
		G/C	1.14 (0.88–1.48)	0.332	0.77 (0.58–1.02)	0.063
		C/C	1.08 (0.71–1.64)	0.713	0.64 (0.39–1.06)	0.084
	Dominant	G/G	1		1	
		G/C-C/C	1.13 (0.88–1.44)	0.345	0.74 (0.57–0.97)	0.031^*^
	recessive	G/G-G/C	1		1	
		C/C	1.02 (0.68–1.52)	0.926	0.73 (0.45–1.18)	0.196
	log-additive	−	1.07 (0.89–1.29)	0.455	0.79 (0.64–0.97)	0.025^*^
rs36045143	Allele	G	1	0.379	1	
		A	1.13 (0.86–1.47)		0.57 (0.42–0.78)	0.000^*^
	codominant	G/G	1		1	
		G/A	1.23 (0.91–1.66)	0.175	0.52 (0.36–0.74)	0.000^*^
		A/A	0.25 (0.05–1.27)	0.095	0.33 (0.06–1.76)	0.194
	dominant	G/G	1		1	
		G/A-A/A	1.17 (0.87–1.57)	0.303	0.51 (0.36–0.72)	0.000^*^
	recessive	G/G-G/A	1		1	
		A/A	0.24 (0.05–1.21)	0.084	0.37 (0.07–1.99)	0.249
	log-additive	−	1.09 (0.82–1.44)	0.550	0.53 (0.38–0.73)	0.000^*^

AR: Allergic rhinitis; SNP: Single nucleotide polymorphism; OR: Odds ratio; CI: Confidence interval.

^*^ Means significant difference.

*p*-value was calculated by logistic regression adjusted by age, sex and BMI.

**TABLE 5:** Association between *CLEC16A* variants and AR risk stratified by sex and BMI.

SNP-ID	Model	Genotype	Male		Female		BMI>24		BMI≤24	
			OR (95% CI)	*p*-value	OR (95% CI)	*p*-value	OR (95% CI)	*p*-value	OR (95% CI)	*p*-value
rs2286973	Allele	A	1	0.960	1	0.052	1	0.092	1	
		G	1.01 (0.81–1.25)		0.85 (0.72–1.00)		1.03 (0.86–1.24)		0.78 (0.65–0.95)	0.012^*^
	Codominant	A/A	1		1		1		1	
		G/A	1.15 (0.69–1.89)	0.573	0.90 (0.72–1.14)	0.401	1.06 (0.81–1.37)	0.686	0.78 (0.59–1.02)	0.066
		G/G	0.91 (0.67–1.25)	0.594	0.65 (0.45–0.95)	0.027^*^	1.03 (0.67–1.57)	0.890	0.62 (0.41–0.95)	0.029^*^
	Dominant	A/A	1		1		1		1	
		G/A-G/G	0.95 (0.71–1.29)	0.760	0.85 (0.68–1.06)	0.151	1.05 (0.82–1.34)	0.696	0.74 (0.57–0.96)	0.023^*^
	Recessive	A/A-G/A	1		1		1		1	
		G/G	1.20 (0.75–1.93)	0.452	0.69 (0.48–0.98)	0.040^*^	1.00 (0.67–1.50)	0.989	0.71 (0.47–1.06)	0.093
	log-additive	−	1.01 (0.81–1.27)	0.990	0.84 (0.71–0.99)	0.041^*^	1.03 (0.85–1.24)	0.763	0.78 (0.65–0.95)	0.013^*^
rs887864	Allele	G	1	0.694	1	0.023^*^	1	0.617	1	
		A	0.95 (0.75–1.22)		0.81 (0.67–0.97)		0.95 (0.77–1.17)		0.77 (0.62–0.95)	0.014^*^
	Codominant	G/G	1	0.404	1	0.104	1	0.594	1	
		G/A	0.62 (0.31–1.22)		0.82 (0.65–1.04)		1.07 (0.83–1.39)		0.80 (0.61–1.04)	0.099
		A/A	1.14 (0.84–1.56)	0.164	0.59 (0.35–0.99)	0.044^*^	0.67 (0.38–1.18)	0.167	0.53 (0.29–0.96)	0.037^*^
	Dominant	G/G	1	0.722	1	0.040^*^	1	0.930	1	
		G/A-A/A	1.06 (0.78–1.42)		0.79 (0.63–0.99)		1.01 (0.79–1.3)		0.76 (0.59–0.98)	0.035^*^
	Recessive	G/G-G/A	1	0.117	1	0.078	1	0.135	1	
		A/A	0.59 (0.30–1.14)		0.63 (0.38–1.05)		0.65 (0.37–1.14)		0.58 (0.32–1.04)	0.066
	log-additive	−	0.96 (0.75–1.23)	0.760	0.80 (0.66–0.96)	0.018^*^	0.95 (0.77–1.17)	0.624	0.77 (0.62–0.95)	0.015^*^
rs12935657	Allele	A	1	0.126	1	0.651	1	0.933	1	
		G	0.76 (0.53–1.08)		0.94 (0.73–1.21)		0.99 (0.74–1.32)		0.77 (0.57–1.03)	0.077
	Codominant	A/A	1	0.403	1	0.828	1	0.590	1	
		G/A	0.85 (0.58–1.25)		1.03 (0.78–1.36)		1.09 (0.79–1.50)		0.86 (0.62–1.19)	0.358
		G/G	-	-	0.35 (0.09–1.29)	0.114	0.46 (0.12–1.81)	0.269	-	-
	Dominant	A/A	1	0.256	1	0.913	1	0.770	1	
		G/A-G/G	0.80 (0.55–1.18)		0.98 (0.75–1.30)		1.05 (0.77–1.43)		0.81 (0.59–1.11)	0.193
	Recessive	A/A-G/A	1	−	1	0.111	1	0.259	1	
		G/G	−		0.34 (0.09–1.28)		0.46 (0.12–1.78)		−	−
	log-additive	−	0.76 (0.53–1.10)	0.146	0.76 (0.53–1.10)	0.146	1.00 (0.75–1.34)	0.997	0.77 (0.57–1.04)	0.088
rs11645657	Allele	G	1	0.295	1	0.038^*^	1	0.772	1	
		C	1.13 (0.90–1.42)		0.84 (0.71–0.99)		1.03 (0.85–1.24)		0.83 (0.69–1.01)	0.066
	Codominant	G/G	1	0.403	1	0.263	1	0.397	1	
		G/C	1.29 (0.77–2.15)		0.88 (0.69–1.10)		1.12 (0.86–1.45)		0.81 (0.62–1.06)	0.123
		C/C	1.11 (0.81–1.52)	0.999	0.67 (0.45–0.99)	0.044^*^	0.96 (0.63–1.47)	0.856	0.73 (0.46–1.15)	0.174
	Dominant	G/G	1	0.372	1	0.104	1	0.510	1	
		G/C-C/C	1.14 (0.85–1.53)		0.83 (0.67–1.04)		1.09 (0.85–1.39)		0.80 (0.62–1.03)	0.077
	Recessive	G/G-G/C	1	0.417	1	0.076	1	0.670	1	
		C/C	1.23 (0.75–2.01)		0.71 (0.49–1.04)		0.91 (0.67–1.38)		0.81 (0.52–1.25)	0.333
	log-additive	−	1.13 (0.90–1.40)	0.299	0.84 (0.71–0.99)	0.041^*^	1.03 (0.86–1.24)	0.760	0.84 (0.69–1.02)	0.074
rs36045143	Allele	G	1	0.182	1	0.248	1	0.898	1	
		A	0.79 (0.56–1.12)		0.86 (0.68–1.11)		0.98 (0.74–1.30)		0.71 (0.53–0.94)	0.018^*^
	Codominant	G/G	1	0.509	1	0.557	1	0.982	1	
		G/A	0.31 (0.03–3.03)		0.92 (0.70–1.21)		1.00 (0.74–1.36)		0.79 (0.57–1.08)	0.136
		A/A	0.83 (0.57–1.20)	0.338	0.34 (0.09–1.27)	0.109	0.86 (0.23–3.22)	0.819	−	-
	Dominant	G/G	1	0.250	1	0.376	1	0.984	1	
		G/A-A/A	0.81 (0.56–1.16)		0.89 (0.68–1.16)		1.00 (0.74–1.35)		0.74 (0.54–1.01)	0.057
	Recessive	G/G-G/A	1	0.334	1	0.114	1	0.818	1	
		A/A	0.33 (0.03–3.16)		0.35 (0.09–1.29)		0.86 (0.23–3.21)		−	-
	log-additive	−	0.79 (0.56–1.13)	0.200	0.86 (0.67–1.10)	0.234	0.99 (0.75–1.31)	0.945	0.70 (0.52–0.95)	0.020^*^

AR: Allergic rhinitis; SNP: Single nucleotide polymorphism; OR: Odds ratio; CI: Confidence interval.

^*^ Means significant difference.

*p*-value was calculated by logistic regression adjusted by age, sex and BMI.

In Table 5, the sex stratification displayed that rs2286973, rs887864 and rs11645657 were related to AR risk in females (rs2286973: codominant-G/G genotype: *p* = 0.027; recessive-G/G genotype: *p* = 0.040; log-additive: *p* = 0.041; rs887864: allele-A: *p* = 0.023; codominant-A/A genotype: *p* = 0.044; dominant-G/A-A/A genotype: *p* = 0.040; log-additive: *p* = 0.018; rs11645657: allele-C: *p* = 0.038; codominant-C/C genotype: *p* = 0.044; log-additive: *p* = 0.025), but these loci had no significant association with AR risk in males. The BMI stratification demonstrated that rs2286973, rs11645657 and rs36045143 were correlated with AR risk in the people with BMI≤24 (rs2286973: allele-G: *p* = 0.012; codominant-G/G genotype: *p* = 0.029; dominant-G/A-G/G genotype: *p* = 0.023; log-additive: *p* = 0.013; rs887864: allele-A: *p* = 0.014; codominant-A/A genotype: *p* = 0.037; dominant-G/A-A/A genotype: *p* = 0.035; log-additive: *p* = 0.015; rs36045143: allele-A: *p* = 0.018; log-additive: *p* = 0.020). Whereas, these SNPs were not found to be associated with AR risk in the people with BMI>24.

### Effect of *CLEC16A* variants on AR risk stratified by region

Meanwhile, we evaluated the association between *CLEC16A* variants and AR risk stratified by region (wind beach area and loess hilly area) in Table 6. In the people from loess hilly area, rs887864 conferred a reduced risk to AR in the codominant (A/A genotype: *p* = 0.015) and recessive (A/A genotype: *p* = 0.016) models. Yet, the site was not found to be associated with the risk of AR in the people from wind beach area.

**TABLE 6:** Association between *CLEC16A* variants and AR risk stratified by region.

SNP-ID	Model	Genotype	Wind beach area		Loess hilly area	
			OR (95% CI)	*p*-value	OR (95% CI)	*p*-value
rs2286973	Allele	A	1	0.718	1	
		G	0.95 (0.74–1.23)		0.88 (0.76–1.03)	0.120
	codominant	A/A	1	0.203	1	
		G/A	0.79 (0.54–1.14)		0.96 (0.77–1.20)	0.729
		G/G	1.01 (0.57–1.77)	0.984	0.72 (0.50–1.03)	0.070
	dominant	A/A	1	0.291	1	
		G/A-G/G	0.83 (0.58–1.18)		0.91 (0.74–1.12)	0.378
	recessive	A/A-G/A	1	0.622	1	
		G/G	1.14 (0.67–1.94)		0.73 (0.52–1.03)	0.073
	log-additive	−	0.93 (0.72–1.21)	0.591	0.89 (0.76–1.04)	0.134
rs887864	Allele	G	1	0.461	1	
		A	0.90 (0.68–1.19)		0.84 (0.71–1.00)	0.051
	codominant	G/G	1	0.497	1	
		G/A	0.88 (0.62–1.27)		0.95 (0.76–1.18)	0.632
		A/A	0.76 (0.33–1.75)	0.526	0.55 (0.34–0.89)	0.015^*^
	dominant	G/G	1	0.428	1	
		G/A-A/A	0.87 (0.61–1.23)		0.88 (0.72–1.09)	0.246
	recessive	G/G-G/A	1	0.599	1	
		A/A	0.80 (0.35–1.82)		0.56 (0.35–0.90)	0.016^*^
	log-additive	−	0.88 (0.66–1.18)	0.391	0.85 (0.71–1.01)	0.061
rs12935657	Allele	A	1	0.488	1	
		G	0.87 (0.59–1.29)		0.87 (0.69–1.11)	0.278
	codominant	A/A	1	1.000	1	
		G/A	1.00 (0.65–1.54)		0.97 (0.74–1.26)	0.812
		G/G	−	−	0.30 (0.08–1.08)	0.066
	dominant	A/A	1	0.790	1	
		G/A-G/G	0.94 (0.62–1.45)		0.92 (0.71–1.20)	0.542
	recessive	A/A-G/A	1	−	1	
		G/G	−		0.30 (0.08–1.09)	0.067
	log-additive	−	0.89 (0.59–1.34)	0.568	0.88 (0.69–1.13)	0.317
rs11645657	Allele	G	1	0.934	1	
		C	0.99 (0.76–1.28)		0.91 (0.78–1.06)	0.236
	codominant	G/G	1	0.764	1	
		G/C	0.95 (0.66–1.36)		0.96 (0.77–1.19)	0.704
		C/C	1.03 (0.58–1.81)	0.922	0.77 (0.53–1.12)	0.173
	dominant	G/G	1	0.830	1	
		G/C-C/C	0.96 (0.68–1.36)		0.92 (0.75–1.13)	0.442
	recessive	G/G-G/C	1	0.844	1	
		C/C	1.06 (0.62–1.81)		0.79 (0.55–1.13)	0.191
	log-additive	−	0.99 (0.77–1.28)	0.948	0.91 (0.78–1.07)	0.242
rs36045143	Allele	G	1	0.558	1	
		A	0.89 (0.61–1.31)		0.82 (0.65–1.03)	0.093
	codominant	G/G	1	0.983	1	
		G/A	1.00 (0.65–1.52)		0.86 (0.66–1.11)	0.243
		A/A	−	−	0.44 (0.13–1.43)	0.171
	dominant	G/G	1	0.800	1	
		G/A-A/A	0.95 (0.62–1.44)		0.83 (0.65–1.08)	0.162
	recessive	G/G-G/A	1	−	1	
		A/A	−		0.45 (0.14–1.47)	0.187
	log-additive	−	0.90 (0.60–1.34)	0.596	0.82 (0.65–1.05)	0.109

AR: Allergic rhinitis; SNP: Single nucleotide polymorphism; OR: Odds ratio; CI: Confidence interval.

^*^ Means significant difference.

*p*-value was calculated by logistic regression adjusted by age, sex and BMI.

### SNP-SNP interaction was used to predict AR risk

**FIGURE 1:**
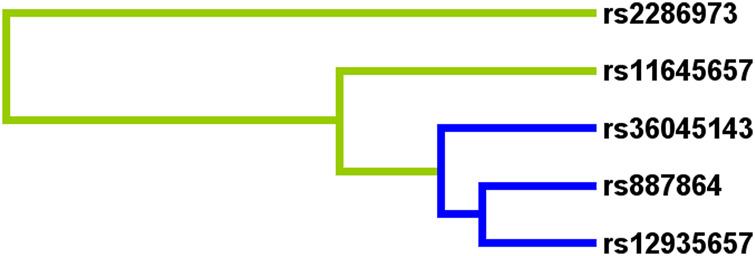
The interaction between rs2286973, rs887864, rs12935657, rs11645657 and rs36045143 were shown in the dendrogram. Antagonism was indicated by blue, and green.

**FIGURE 2:**
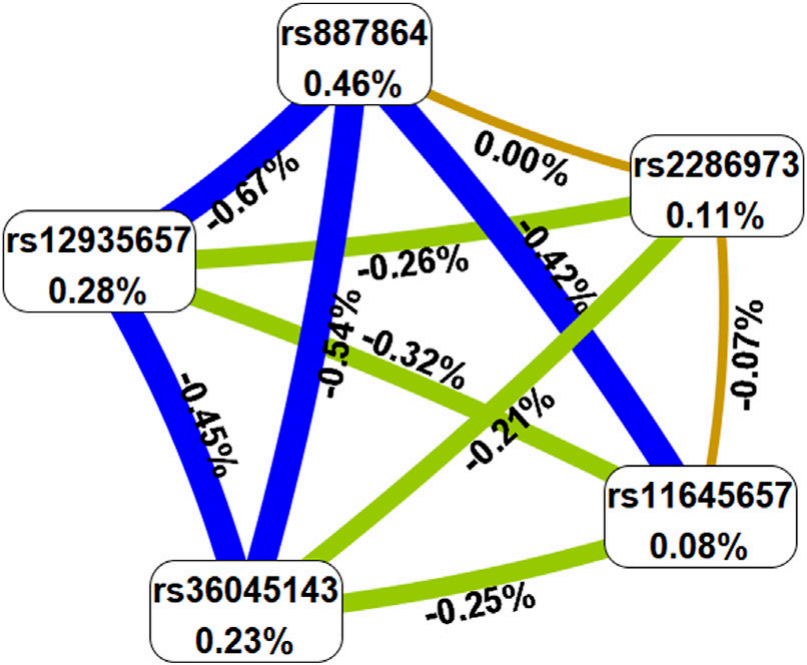
The circle graph showed that the interaction between rs2286973 and rs887864 was the strongest, with the information gain (IG) value 0.00%. Antagonism was indicated by blue, green and brown.

To predict the AR risk, we did the SNP-SNP interaction among *CLEC16A* variants by MDR method (Supplementary Table S5, Figure 1 and Figure 2). In the Supplementary Table S5, the best model was composed of rs2286973 G/A, rs887864 G/A, rs12935657 G/A, rs11645657 C/G and rs36045143 A/G, with cross-validation consistency (CVC) of 10/10 and testing balanced accuracy of 55% (adjusted OR: 2.05, 95%CI: 1.67–2.51, *p* < 0.000). In the Supplementary Table S6, we have listed all possible genotype combinations. Among them, the high-risk genotypes (ratio greater than 1) were AG*GA*GG*CC*AA, AG*GA*GG*GC*AA, AG*GA*GA*GC*AG, AA*GA*GA*GG*AG, AA*GA*GA*GC*AG,AA*GG*GG*CC*AA, GG*AA*GG*CC*AA and GG*AA*GG*GC*AA. In addition, we showed the interaction between each site with the dendrogram (Figure 1) and the circle graph (Figure 2). Antagonism was indicated by blue, green and brown. In Figure 2, the interaction between rs2286973 and rs887864 was the strongest, with the information gain (IG) value 0.00%.

## Discussion

*CLEC16A* is found in the 16p13 area next to *CIITA* gene. It was reported that CLEC16A lacks a functional Ag recognition domain, and Schuster et al. observed that it can induce autoimmune responses in mice, possibly by stimulating Ag expression in thymic epithelial cells (Schuster et al., 2015). More importantly, CLEC16A may be the main regulator of autoimmunity, like Type 1 diabetes (Barrett et al., 2009), primary biliary cirrhosis (Hirschfield et al., 2012), alopecia areata (Jagielska et al., 2012). Also, *CLEC16A* variations were observed to affect the selection and reactivity of T cells (Schuster et al., 2015). The study provided a link between *CLEC16A* variants and immune disorders underlying autoimmune risk. Based on the genome wide association analysis, Ferreira et al. identified the risk variations associated with hay fever phenotype asthma, including the sites of *CLEC16A* (Ferreira et al., 2014)*.* Among them, rs62026376 C allele was observed to be associated with an increased risk of hay fever asthma. In addition, Gao et al. noticed that *CLEC16A* rs7203459 was a specific susceptibility locus of AR in the Han Chinese population (Gao et al., 2020). In our research, we also observed the significant association between *CLEC16A* SNPs (rs2286973, rs887864, rs12935657, rs11645657 and rs36045143) and AR risk. It showed the importance of *CLEC16A* polymorphisms in AR. The analysis of the interaction between rs2286973, rs887864, rs12935657, rs11645657 and rs36045143 is helpful to discover the risk factors of AR. Our results showed that the best model was composed of rs2286973 G/A, rs887864 G/A, rs12935657 G/A, rs11645657 C/G and rs36045143 A/G for AR.

The incidence of AR increased with age (Westman et al., 2012). It has been reported that there is a certain prevalence of AR in childhood (Wise et al., 2018). Once allergic symptoms appear, the symptoms generally persist into adulthood. As much as 80% of AR patients develop symptoms before the age of 20 years (Roditi and Shin, 2018). Not only that, some researchers observed that the influence of some susceptible genes on AR was related to age (Zhang and Xu, 2020; Yin et al., 2021). In this study, we took the average age of 43 as the critical value for stratified analysis. The results showed rs2286973, rs11645657 and rs36045143 were related to a decreased risk of AR in the people ≤43 years old, while no such association was found in people over 43 years of age. These findings suggest that rs2286973, rs11645657 and rs36045143 polymorphisms have age-related effect on AR risk.

AR was a sex-specific disease (Osman et al., 2007). In one article reported by Rosário et al., boys often appeared allergic reactions in childhood, while girls were more likely to develop allergic disorders (including AR) during sexual development (Rosário et al., 2021). This may be related to sex hormones, lifestyle, dietary differences, professional choice and treatment compliance and other factors (Stübner et al., 1999). What’s more, genetic susceptibility to AR varied by sex (Tian et al., 2015). In the present study, rs2286973, rs887864 and rs11645657 were related to AR risk in females, but these loci had no significant association with AR risk in males. The findings reemphasized the importance of sex in the study of the association between genetics and AR risk.

BMI was an influential factor for the high prevalence of AR (Lokaj-Berisha et al., 2015). Not only that, some reporters found that variants of genes were associated with the susceptibility to AR. Wang et al. found that LPP (rs2030519, rs6780858 and rs2990220) were related to AR risk (Wang et al., 2022). Lian et al. observed that Related Orphan Receptor A (RORA)-rs10519067, rs10519068, and rs11071559-were associated with AR patients with BMI ≤24 kg/m^2^ (Lian et al., 2022). In the study, rs2286973, rs11645657 and rs36045143 were correlated with AR risk in the people with BMI≤24, illustrated that AR risk was influenced by BMI.

Regionality was also a factor affecting AR. In our previous research (Gao et al., 2021), the prevalence of asthma without AR was 1.55 times higher in people living in plain areas than that in people living in hilly areas. Moreover, we found that the prevalence of AR with asthma was 2.00 times higher in plains than that in hills. These results indicated that the incidence of asthma and AR was closely related to the place of residence of the patients. In order to further study the impact of the region on AR, we assessed the association between *CLEC16A* variants and AR risk stratified by region (wind beach area and loess hilly area). In the people from loess hilly area, rs887864 conferred a reduced risk to AR. Yet, the site was not found to be associated with the risk of AR in the people from wind beach area. A large number of samples were required for subsequent verification.

There are still some deficiencies in this study. Firstly, the study population focused on the Han population, and different ethnic populations should be collected to verify the results. Secondly, the other risk factors (occupational exposures, working condition, family background, physical activity, etc.) were not included and should be considered for future assessment. Thirdly, we used Regulome DB (http://www.regulomedb.org/) and HaploReg predict the function of SNPs (Table 2). Further theoretical experiments were needed to verify that these sites may affect the risk of AR.

## Conclusion

In a short, *CLEC16A* (rs887864, rs12935657, rs2286973, rs11645657 and rs36045143) conferred a decreased risk to AR in the Chinese population.

## Data Availability

The original contributions presented in the study are publicly available. This data can be found here: https://zenodo.org/7284973.
