# Supplementary material for: CLEC16A variants conferred a decreased risk to allergic rhinitis in the Chinese population
Source: Front Genet. 2022 Dec 12;13:1053761. doi: 10.3389/fgene.2022.1053761 (PMC9801328; doi:10.3389/fgene.2022.1053761)
Supplement: Supplementary file 1 [file Table1.DOCX]

Supplementary Table 1 The primer information of *CLEC16A* variants

| SNP | 1st-PCRP | 2nd-PCRP | UEP_SEQ | Director |
| --- | --- | --- | --- | --- |
| rs2286973 | ACGTTGGATGAATTGCAGTGACAGGGAACG | ACGTTGGATGCTCTGCTCTCTGAACTGTTG | GGTCCAGGCCATCCGGGT | F |
| rs887864 | ACGTTGGATGTCTGCTTAAGCAACACTCTG | ACGTTGGATGCTTACCCCTCAAAACAGTGC | acccCCCCTCAAAACAGTGCCATCATC | R |
| rs12935657 | ACGTTGGATGACTGTGCTGATTTGCACAGG | ACGTTGGATGGGCCAAGTTTCAGCTAAGTG | gatgCAGCTAAGTGAGAAATGAGA | R |
| rs11645657 | ACGTTGGATGCAAGGATTTCCAGACCACAC | AACGTTGGATGTACAGACATCCAGTGCAAGG | cccGCCCTGGGGCAGGGAAG | R |
| rs36045143 | ACGTTGGATGACATACTGGCCGCCACTGA | ACGTTGGATGACCCTCACAGAATGTTCAGC | tccccGAATGTTCAGCTTCACG | F |

SNP, single nucleotide polymorphism; PCRP, polymerase chain reaction primer; UEP, unextended mini sequencing primer; SEQ, sequence.

Supplementary Table 2 The clinical information of controls and cases

| Variable | Mean ± SE | | *p*-value |
| --- | --- | --- | --- |
|  | Control | Case |  |
| Red blood cell distribution width_ SD (RDW_SD, fL) | 43.183±0.11 | 42.773±0.10 | 0.008 |
| Basophil count (BASO, 10^^^9/L) | 0.028±0.00 | 0.030±0.00 | <0.001 |
| Eosinophil count (EO, 10^^^9/L) | 0.122±0.00 | 0.228±0.00 | <0.001 |
| Hemoglobin (HGB, g/L) | 144.606±0.59 | 146.362±0.57 | 0.033 |
| Mean hemoglobin concentration (MCHC, g/L) | 328.134±0.32 | 329.421±0.31 | 0.004 |
| Neutrophil ratio (NEUT_per, %) | 60.875±0.24 | 60.179±0.25 | 0.048 |
| Lymphocyte ratio (LYMPH_per, %) | 31.847±0.23 | 31.034±0.22 | 0.010 |
| Eosinophil ratio (EO_per, %) | 1.893±0.06 | 3.456±0.10 | <0.001 |
| Basophil ratio (BASO_per, %) | 0.443±0.01 | 0.480±0.01 | 0.001 |
| Red blood cell distribution width_CV (RDW_CV, %) | 13.318±0.05 | 13.158±0.04 | 0.013 |
| Fasting blood glucose (FBG, mmol/L) | 4.877±0.04 | 4.714±0.03 | 0.003 |
| Uric Acid (UA, μmol/L) | 283.498±2.56 | 301.573±2.68 | <0.001 |

SE: standard error.

*p*-value was calculated by Student’s t-test.

Supplementary Table 3 The relationship between genotypes at different loci and clinical parameters

| SNP-ID | Variable | Genotype | Mean ± SE | |
| --- | --- | --- | --- | --- |
|  |  |  | Control | Case |
| rs2286973 | Eosinophil count (EO) | A/A | 0.13±0.01 | 0.27±0.03 |
|  |  | G/A | 0.13±0.01 | 0.24±0.01 |
|  |  | G/G | 0.12±0.01 | 0.21±0.01 |
|  |  |  | 0.436 | 0.023^*^ |
| rs11645657 | Hemoglobin (HGB) | G/G | 141.87±1.65 | 145.24±2.12 |
|  |  | G/C | 143.10±0.93 | 146.29±0.84 |
|  |  | C/C | 146.51±0.86 | 146.65±0.84 |
|  |  |  | 0.007^*^ | 0.790 |
|  | Lymphocyte ratio (LYMPH_per) | G/G | 32.86±0.78 | 31.08±0.73 |
|  |  | G/C | 31.89±0.35 | 30.36±0.35 |
|  |  | C/C | 31.59±0.31 | 31.58±0.32 |
|  |  |  | 0.259 | 0.035^*^ |
|  | Uric Acid (UA) | G/G | 276.65±6.99 | 319.90±9.22 |
|  |  | G/C | 277.00±3.95 | 298.27±4.22 |
|  |  | C/C | 290.63±3.79 | 301.18±3.72 |
|  |  |  | 0.028^*^ | 0.087 |

SE, Standard error.

*p*-value﹤0.05 indicate statistical significance (^*^).

Supplementary Table 4 FPRP analysis for the significant associations of *CLEC16A* variants with allergic rhinitis risk

| Model | OR(95%CI) | Power | Prior probability | | | | |
| --- | --- | --- | --- | --- | --- | --- | --- |
|  |  |  | 0.25 | 0.1 | 0.01 | 0.001 | 0.0001 |
| rs887864 |  |  |  |  |  |  |  |
| G vs A | 0.86(0.74-0.99) | 1.000 | 0.097^*^ | 0.243 | 0.780 | 0.973 | 0.997 |
| G/G vs A/A | 0.60(0.40-0.91) | 0.310 | 0.136^*^ | 0.320 | 0.838 | 0.981 | 0.998 |
| G/G-G/A vs A/A | 0.62(0.41-0.93) | 0.363 | 0.147^*^ | 0.341 | 0.850 | 0.983 | 0.998 |
| rs12935657 |  |  |  |  |  |  |  |
| A/A vs G/G | 0.24(0.07-0.83) | 0.053 | 0.576 | 0.803 | 0.978 | 0.998 | 1.000 |
| A/A-G/A vs G/G | 0.24(0.07-0.84) | 0.055 | 0.582 | 0.807 | 0.979 | 0.998 | 1.000 |

FPRP, False-positive report probability.

The level of FPRP threshold was set at 0.2 and noteworthy findings are presented (^*^).

Supplementary Table 5 SNP-SNP interaction of *CLEC16A* variants were analyzed by the MDR method

| Model | Training Bal. Acc. | Testing Bal. Acc. | CVC | OR (95% CI) | *p* |
| --- | --- | --- | --- | --- | --- |
| rs887864 | 0.516 | 0.499 | 5/10 | 1.13(0.95-1.35) | 0.172 |
| rs2286973, rs887864 | 0.538 | 0.532 | 10/10 | 1.53(1.24-1.89) | < 0.000^*^ |
| rs2286973, rs887864, rs11645657 | 0.558 | 0.548 | 10/10 | 1.79(1.47-2.19) | < 0.000^*^ |
| rs2286973, rs887864, rs11645657, rs36045143 | 0.565 | 0.543 | 8/10 | 1.98(1.61-2.44) | < 0.000^*^ |
| rs2286973, rs887864, rs12935657, rs11645657, rs36045143 | 0.569 | 0.550 | 10/10 | 2.05(1.67-2.51) | < 0.000^*^ |

MDR, multifactor dimensionality reduction; Bal. Acc., balanced accuracy; CVC, cross–validation consistency; OR, odds ratio; CI, confidence interval.

*p* values were calculated using χ^2^ tests.

*p* < 0.05 indicates statistical significance (^*^).

Supplementary Table 6 Possible genotype combinations

| Combination | Case(N) | Control(N) | Ratio |
| --- | --- | --- | --- |
| AG,GA,GG,CC,AA | 132 | 117 | 1.1282 |
| AG,GA,GG,CC,AG | 1 | 2 | 0.5 |
| AG,GA,GG,GG,AA | 4 | 2 | 2 |
| AG,GA,GG,GG,AG | 0 | 2 | 0 |
| AG,GA,GG,GC,AA | 31 | 30 | 1.0333 |
| AG,GA,GG,GC,AG | 5 | 10 | 0.5 |
| AG,GA,GA,GG,AA | 1 | 0 | ∞ |
| AG,GA,GA,GG,AG | 22 | 32 | 0.6875 |
| AG,GA,GA,GC,GG | 1 | 0 | ∞ |
| AG,GA,GA,GC,AG | 104 | 92 | 1.1304 |
| AG,GG,GG,CC,AA | 0 | 5 | 0 |
| AG,GG,AA,GG,GG | 0 | 2 | 0 |
| AG,GG,GA,GC,AG | 0 | 7 | 0 |
| AG,AA,GG,CC,AA | 41 | 57 | 0.7193 |
| AG,AA,GG,CC,AG | 1 | 1 | 1 |
| AG,AA,GG,GG,AA | 24 | 12 | 2 |
| AG,AA,GG,GC,AA | 61 | 82 | 0.7439 |
| AG,AA,GG,GC,AG | 1 | 0 | ∞ |
| AG,AA,GA,GG,AG | 2 | 0 | ∞ |
| AG,AA,GA,GC,AA | 1 | 1 | 1 |
| AG,AA,GA,GC,AG | 2 | 0 | ∞ |
| AA,GA,GG,CC,AA | 11 | 15 | 0.7333 |
| AA,GA,GG,GG,AA | 1 | 1 | 1 |
| AA,GA,GG,GG,AG | 1 | 0 | ∞ |
| AA,GA,GG,GC,AA | 10 | 13 | 0.7692 |
| AA,GA,GG,GC,AG | 1 | 2 | 0.5 |
| AA,GA,AA,GG,GG | 0 | 1 | 0 |
| AA,GA,AA,GG,AG | 1 | 0 | ∞ |
| AA,GA,GA,GG,AA | 0 | 1 | 0 |
| AA,GA,GA,GG,AG | 13 | 7 | 1.8571 |
| AA,GA,GA,GC,AG | 7 | 5 | 1.4 |
| AA,GG,GG,CC,AA | 15 | 8 | 1.875 |
| AA,GG,GG,GC,AA | 3 | 3 | 1 |
| AA,GG,GG,GC,AG | 0 | 2 | 0 |
| AA,GG,AA,GG,GG | 2 | 8 | 0.25 |
| AA,GG,GA,GG,GG | 0 | 1 | 0 |
| AA,GG,GA,GG,AG | 0 | 1 | 0 |
| AA,GG,GA,GC,AG | 18 | 22 | 0.8182 |
| AA,AA,GG,CC,AA | 4 | 8 | 0.5 |
| AA,AA,GG,GG,AA | 0 | 6 | 0 |
| AA,AA,GG,GC,AA | 10 | 10 | 1 |
| GG,GA,GG,CC,AA | 1 | 8 | 0.125 |
| GG,GA,GG,CC,AG | 1 | 1 | 1 |
| GG,GA,GG,GG,AA | 1 | 1 | 1 |
| GG,GA,GG,GG,AG | 0 | 1 | 0 |
| GG,GA,GG,GC,AA | 3 | 2 | 1.5 |
| GG,GA,GG,GC,AG | 0 | 1 | 0 |
| GG,GA,GA,GG,AG | 0 | 4 | 0 |
| GG,GA,GA,GC,AG | 1 | 11 | 0.0909 |
| GG,GG,GG,CC,AA | 0 | 1 | 0 |
| GG,GG,GA,GC,AG | 0 | 3 | 0 |
| GG,AA,GG,CC,AA | 278 | 254 | 1.0945 |
| GG,AA,GG,CC,AG | 1 | 0 | ∞ |
| GG,AA,GG,GG,AA | 14 | 18 | 0.7778 |
| GG,AA,GG,GC,AA | 137 | 117 | 1.1709 |
| GG,AA,GA,CC,AA | 1 | 0 | ∞ |
| GG,AA,GA,GG,AG | 1 | 0 | ∞ |
| GG,AA,GA,GC,AA | 1 | 0 | ∞ |
| GG,AA,GA,GC,AG | 6 | 0 | ∞ |
